# On Porous Rarefaction of the Bone, Consequent upon Gout

**Published:** 1847-09

**Authors:** Alexander Ure

**Affiliations:** Fellow of the Royal College of Surgeons, and Surgeon to the Westminster General Dispensary


*On Porous Rarefaction of the Bone, consequent upon gout.* By ALEXANDER URE, Esq., Fellow of the Royal College of Surgeons, and Surgeon to the Westminster General Dispensary.—The author observed that, upon making a transverse section of a digital phalanx, taken from a gouty subject, a peculiar speckled appearance was perceptible. The medullary canals were seen preternaturally enlarged, and filled with a cretaceous matter, which effervesced with acids. The Haversian canals were, in like manner, irregularly enlarged and choked up with this substance. The osseous lacunæ or corpuscles were, in some places, increased in size, and rounder than ordinary, but, for the most part, less distinctly marked. The canaliculi, more especially in the vicinity of the medullary canals, were replete with the above deposit, thicker than normal, and in obvious communication with the medullary canals. There was thus induced porous rarefaction of the bone. The author stated that the matter in question consisted chiefly of carbonate of lime, while a portion of tophus, detached from the adjunct phalangar joint, was almost wholly composed of urate of soda. In reference to the above peculiar condition of the osseous structure, he remarked, that Dr. Gerlach, of Mayence, whose co-operation he had in the above research, subsequently ascertained that precisely the same appearances were present in the section of bone taken from a person whohad been afflicted with morbus coxæ senilis. Arthritic osteoporosis is an affection of a very insidious character. It commences almost imperceptibly, creeps on stealthily, year by year, and is accompanied by occasional pain and swelling Although usually occurring in individuals hereditarily predisposed to gout, it is not necessarily preceded by a fit of that malady. While the author believes it to be but little amenable to treatment, he is of opinion its progress may be somewhat retarded by judicious hygienic measures; and the attendant pain and uneasiness relieved by topical steam baths, in conjunction with the vapour of mineral naphtha, and by various other soothing means.—*Ib.*

